# Bilateral Quadriceps Tendon Rupture in a Healthy Individual Following a Motor Vehicle Accident: A Case Report

**DOI:** 10.7759/cureus.36245

**Published:** 2023-03-16

**Authors:** Joe Ghanimeh, Anthony El Alam, Joeffroy Otayek, Alfred Khoury

**Affiliations:** 1 Orthopedics and Traumatology, Lebanese American University Medical Center, Beirut, LBN

**Keywords:** anchor suture repair, post-operative care, case report, healthy individual, bilateral quadriceps tendon rupture

## Abstract

Bilateral quadriceps tendon rupture (QTR) is a rare injury that typically affects middle-aged men presenting underlying medical conditions, while only a few cases have been reported in healthy individuals. The gold standard treatment of such injuries is prompt surgical repair, followed by postoperative immobilization and physiotherapy.

We present the case of a 51-year-old previously healthy man who experienced bilateral, simultaneous, and complete QTR following a high-velocity motor vehicle accident. Physical examination revealed bilateral extensor mechanism disruption and palpable defects at the superior poles of the patellae. MRI confirmed the diagnosis, and the patient underwent surgical repair using three anchor sutures on each side. Postoperative management involved a brief period of immobilization followed by progressive passive motion exercises and protected weight bearing. At a six-month follow-up, the patient had excellent functional outcomes and was satisfied with the treatment.

## Introduction

Bilateral quadriceps tendon rupture (QTR) is a rare injury that typically occurs in male patients with underlying medical conditions, including chronic kidney failure, hyperparathyroidism, systemic lupus erythematosus, rheumatoid arthritis, diabetes, obesity, and gout. Fluoroquinolone and corticosteroid use and anabolic steroid use have also been identified as additional risk factors for QTR [1-3].

Some of these predisposing conditions are thought to induce pathologic tendon degeneration, making it prone to rupture, even in the context of a minor trauma such as a stumbling or a simple fall [2,3]. However, rupture is still possible in healthy individuals, especially following violent eccentric contractions of the quadriceps, or during forced knee hyperflexion [2,4,5].

In active individuals, surgical repair of the extensor mechanism is the gold standard treatment, which can involve a range of techniques such as transosseous (TO) sutures, suture anchors (SAs), and knotless anchor suture repair [6]. Delayed repair is more challenging due to soft tissue retraction and fibrosis and might need a more complex procedure such as the use of grafts or muscle transfers [7]. Postoperative care usually involves a variable period of immobilization and physiotherapy, but there is no clear consensus on the optimal protocol [7,8].

Here, we present the case of a 51-year-old previously healthy man who sustained bilateral QTR following a high-velocity motor vehicle accident.

## Case presentation

We received a 51-year-old male patient of East-African ethnicity who had been in a motor vehicle accident three days prior to presentation.The patient had no known past medical or surgical history and reported taking no medications at home, including quinolones and steroids. He did not experience any symptoms or knee-related complaints prior to the accident and was able to perform all activities of daily life with no limitations.

During the accident, the patient was riding a motorcycle and was projected two meters from his vehicle, landing on the ground with direct trauma to both his knees in a hyperflexed position. He was brought to our department due to bilateral knee pain and an inability to bear weight.

Upon physical examination, we observed a highly muscular patient who weighed 130 kg and measured 192 cm in height, resulting in a BMI of 35.3. The patient had normal sensation in both lower extremities with normal peripheral pulses. Lower extremities motor power was globally preserved, except for an inability to perform an active straight leg raise bilaterally. A bilateral knee swelling was noted along a palpable defect at the proximal patellar poles.

**Figure 1:**
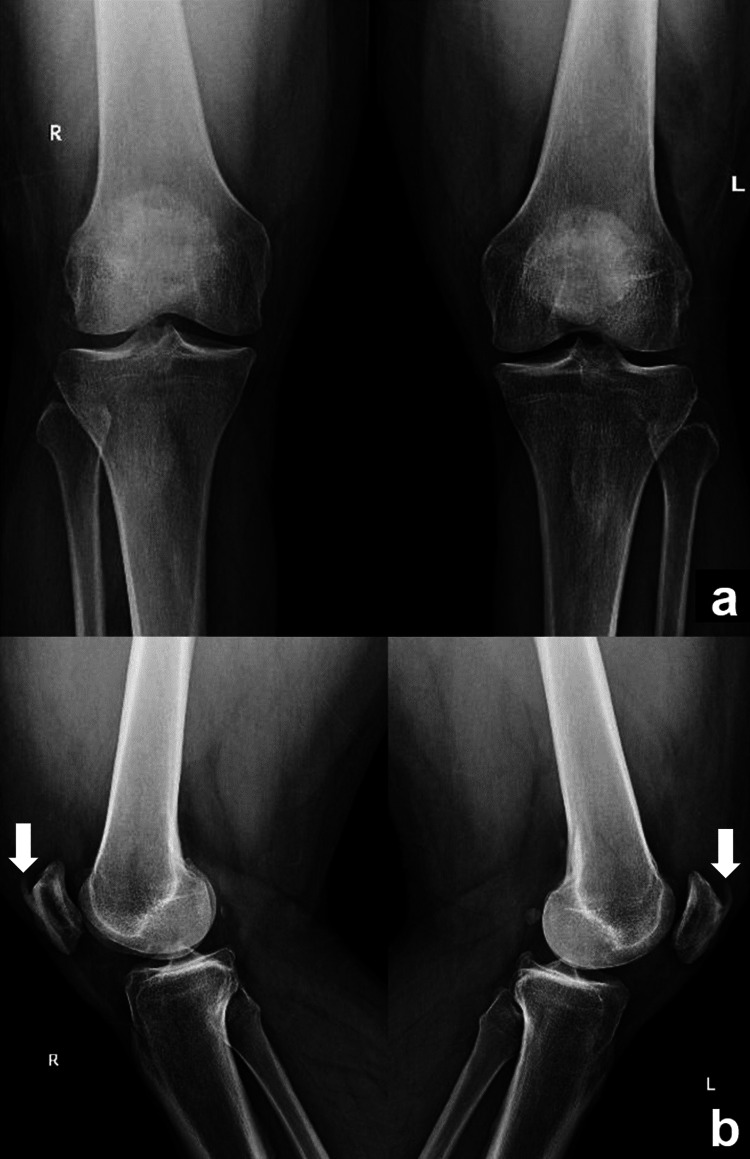
Bilateral knee anteroposterior (a) and lateral (b) radiographic views. White arrows indicate the bony spurs (enthesophytes) at the superior poles of the patellae

Upon admission, complete blood count, uric acid, chemistry (Chem9), and inflammatory markers were negative. Bilateral knee radiographs showed normal osseous structures and alignment with mild degenerative changes. We also observed enthesophyte formation at the insertion of the quadriceps tendon on the superior patella bilaterally (see Figure 1).

**Figure 2:**
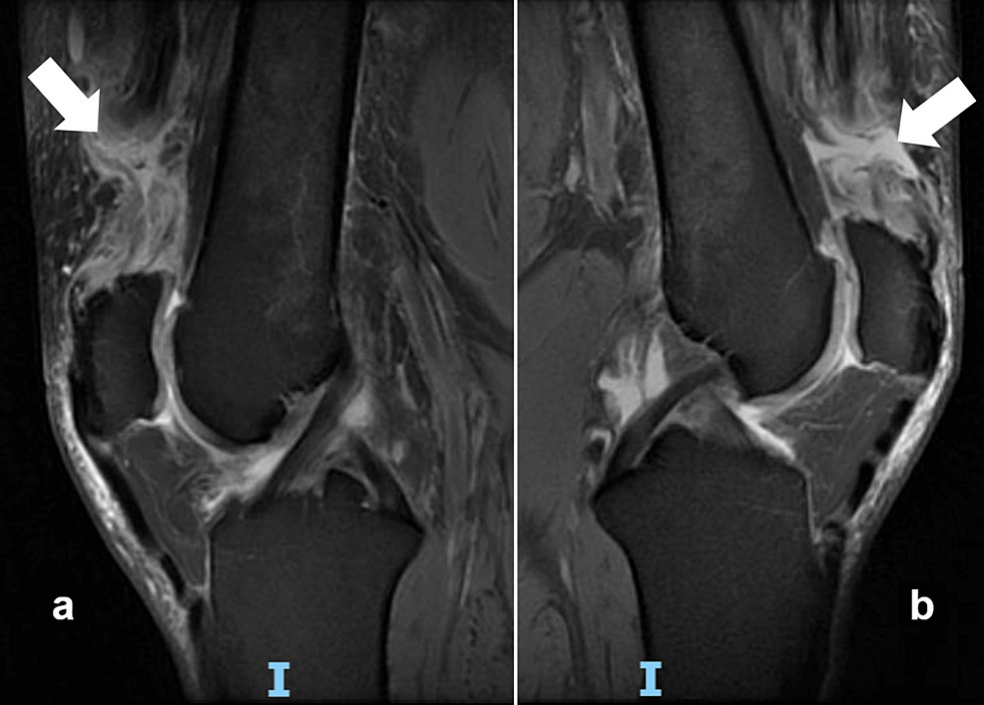
T2 MRI sagittal cuts of the right (a) and left (b) knees. White arrows indicate the site of rupture for each quadriceps tendon.

MRI was performed for both knees and confirmed the diagnosis of complete bilateral QTR. The lesion occurred at 22 mm from the tendon insertion on the left side and at 35 mm from the insertion on the right side, with thickening and edema of the retracted tendons stumps both proximally and distally (Figure 2).

The patient was diagnosed with a bilateral traumatic QTR and was taken to the operating room for surgical management. Under spinal anesthesia and tourniquets at 350mmhg, two teams operated simultaneously using midline incisions to expose the superior patellar poles and ruptured tendons. Debridement of the tendons edges was performed, followed by preparation of each patella to receive three 5.5 mm Healix advanced anchor sutures (DePuy Synthes, Johnson & Johnson). Reinsertion of the proximal ends was done using Karckow sutures and reinforced with Vicryl sutures. Absorbable sutures and staplers were used for skin closure.

In the immediate postoperative phase, both legs were immobilized in extension using a knee brace, and ice therapy was applied bilaterally. The patient was discharged on day two postoperatively, pain-free, and started on Lovenox (low molecular weight heparin 40mg subcutaneously daily). Gentle continuous passive motion (CPM) was initiated on day fourteen postoperatively, starting at 0-30 degrees and increasing by 10 degrees weekly. Ambulation with two crutches and protected weight bearing was also started on day fourteen. Follow-up at four weeks postoperatively showed a healed wound and good range of motion at 0-50 degrees. Active extension was not allowed before six weeks postoperatively. At the last follow-up, six months postoperatively, the patient had a near-baseline range of motion and reported excellent satisfaction.

## Discussion

The first reported case of bilateral QTR was described in 1949 by Steiner and Palmer in a 67-year-old, obese, white male [9]. Only 105 cases were then reported during the following 55 years, accounting for an average of less than two cases per year [3].

The specificity of the present case resides in the absence of pre-existing comorbidities that could have induced this injury. The fact that most patients with bilateral QTR have multiple risk factors makes its occurrence in a healthy individual even more unusual [1,3]. A recent study by Panagopoulos et al. yielded a total of only 14 cases of bilateral QTR in healthy patients in English literature, in addition to one case who failed surgical treatment and needed reoperation [10]. All 15 patients were males with a mean age of 56 years. Though the demographic characteristics of our patient go in line with those of this study, our patient’s high-velocity mechanism contrasts with the milder trauma sustained by all patients, of whom none was a victim of MVA. The likelihood of sustaining a bilateral QTR during an MVA appears to be excessively low, as no patient presented with this mechanism in another review of 30 cases by Neubauer et al. [3].

Yepes et al. divided the quadriceps tendon into three zones: Zone 1 between 0 and 1cm from the patellar insertion, zone 2 between 1 and 2 cm, and zone 3 at >2cm from the patella [11]. Both lesions in the present case occurred in zone 3, more obviously on the right side, which contrasts with the usual location of these lesions (35.6% in zone 1, 41.4% in zone 2, and only 12.1% in zone 3) [8].

While the present case did not have previous medical conditions or any knee-related complaints, it is worth mentioning the insertional enthesophytes at the superior poles of his patellas. The incidence of such spurs at the quadriceps-patella junction ranges from 62% to 79% in patients presenting a quadriceps rupture versus 19% in the general population [12,13]. Although a clear causative relationship is not clearly established, such findings on knee x-rays of patients with knee trauma should raise the suspicion of a QTR and warrant further investigations.

The surgical team opted for a SA repair for this particular case instead of the commonly used TO suture technique. This decision was based on medical literature that compared the two methods. Several cadaveric studies have shown that SA repair is superior in terms of gapping during cyclic loading [14,15], although there were no significant differences in the ultimate load to failure [16]. Clinical studies have also reported better patient-reported outcomes for SA repair [17], while others found no significant differences in functional outcomes between the two techniques [18]. Therefore, SA repair appeared to us to be at least as effective as TO repair while avoiding the need for further dissection to expose all the patella, as well as drilling tunnels inside it, which can be more challenging and might increase the risk of patellar fracture.

Moreover, delay between time of injury and surgery was minimized as much as possible, as recent studies highlighted the benefits of early repair compared to delayed treatment in terms of patient satisfaction, healing potential, and restoration of near-normal function of the extensor mechanism, regardless of the surgical modality [8,19].

Traditional postoperative care for QTR included immobilization in extension to allow tendon healing prior to resuming motion, with most authors advocating for a six-week period [7,8,10]. Some authors experimented earlier return to the passive range of motion in an effort to reduce complications related to bed rest and limit muscle atrophy post immobilization. In these trials, time to return to passive range of motion ranged from two days to three weeks with comparable results in longer immobilization periods [19,20]. Though a consensus is yet to be found, in our experience, two weeks of immobilization prior to CPM appear to be a reasonable compromise to allow some tissue healing while avoiding stiffness and preserving quadriceps muscle mass which is a synonym for faster recovery and return to full ROM.

## Conclusions

Bilateral QTR is a rare but serious injury that can cause significant impairment if not treated promptly. Although the optimal surgical and postoperative management approaches are still under discussion, early diagnosis and immediate surgical intervention are crucial for achieving satisfactory functional outcomes. Therefore, clinicians should be aware of the possibility of bilateral QTR in previously healthy individuals sustaining a high-velocity trauma. A high index of suspicion should be maintained when the patient presents with bilateral knee pain, a palpable defect at the quadriceps insertion, and an inability to perform an active straight leg raise, especially if radiographic studies reveal the presence of superior patellar spurs. By recognizing the signs and symptoms of this condition and providing timely treatment, healthcare providers can help patients recover successfully and reduce the risk of long-term disability.
